# Statin Treatment as a Targeted Therapy for APC-Mutated Colorectal Cancer

**DOI:** 10.3389/fonc.2022.880552

**Published:** 2022-05-30

**Authors:** Hannah Shailes, Wai Yiu Tse, Marta O. Freitas, Andrew Silver, Sarah A. Martin

**Affiliations:** ^1^Centre for Cancer Cell and Molecular Biology, Barts Cancer Institute, Queen Mary University of London, London, United Kingdom; ^2^Centre for Genomics and Child Health, Blizard Institute, Barts and the London School of Medicine and Dentistry, Queen Mary University of London, London, United Kingdom

**Keywords:** colorectal (colon) cancer, APC, synthetic lethality, personalized medicine, statin (HMG-CoA reductase inhibitor), biomarker

## Abstract

**Background:**

Mutations in the tumor suppressor gene Adenomatous Polyposis Coli (*APC*) are found in 80% of sporadic colorectal cancer (CRC) tumors and are also responsible for the inherited form of CRC, Familial adenomatous polyposis (FAP).

**Methods:**

To identify novel therapeutic strategies for the treatment of *APC* mutated CRC, we generated a drug screening platform that incorporates a human cellular model of *APC* mutant CRC using CRISPR-cas9 gene editing and performed an FDA-approved drug screen targeting over 1000 compounds.

**Results:**

We have identified the group of HMG-CoA Reductase (HMGCR) inhibitors known as statins, which cause a significantly greater loss in cell viability in the *APC* mutated cell lines and in *in vivo APC* mutated patient derived xenograft (PDX) models, compared to wild-type *APC* cells. Mechanistically, our data reveals this new synthetic lethal relationship is a consequence of decreased Wnt signalling and, ultimately, a reduction in the level of expression of the anti-apoptotic protein Survivin, upon statin treatment in the *APC*-mutant cells only. This mechanism acts *via* a Rac1 mediated control of beta-catenin.

**Conclusion:**

Significantly, we have identified a novel synthetic lethal dependence between *APC* mutations and statin treatment, which could potentially be exploited for the treatment of *APC* mutated cancers.

## Introduction

Colorectal cancer (CRC) is one of the leading causes of cancer deaths in the UK. CRC is a highly heterogeneous disease with diverse genetic and clinical features that influence therapeutic outcomes. Truncation mutations in the Adenomatous Polyposis Coli (APC) gene are found in the inherited syndrome familial adenomatous polyposis (FAP) and in more than 80% of sporadic colon tumors (1–3). These mutations are the initiating events in CRC tumorigenesis, through the activation of the Wnt signaling pathway (4).

*APC* is a tumor suppressor gene and exerts its anti-proliferative effects as an antagonist of the Wnt pathway (4), whereby APC downregulates β-catenin through its association with the APC/Axin/GSK3-β destruction complex. Loss of APC leads to the inappropriate stabilization of β-catenin, which acts as co-activator of transcription with the TCF/LEF transcription factors (5, 6). Transcriptional targets of beta-catenin/TCF include oncogenic genes such as c-myc and cyclin D1. This suggests that deregulation of the Wnt pathway occurs through mutations in APC thereby promoting tumorigenesis. APC mutations occur typically within or around the mutation cluster region and result in a truncated inactive APC protein, as a consequence of either nonsense point mutations or frameshift mutations (7). The ‘just-right’ Wnt signaling hypothesis proposes that different APC mutations provide cells with different selective advantages, determined by the optimal level of Wnt activation necessary for tumorigenesis (8). In mice, the position of an APC mutation can affect polyp number, location, and stage (9, 10). For example, *Apc^Δ242^* mice can suppress intestinal tumorigenesis (11), *Apc^Δ716^* mice develop increased number of polyps, while a more distal mutation, *Apc^1634N^*, results in greatly reduced tumor burden (12, 13). These data recapitulate findings from FAP patients in which disease severity can correlate with the location of the germline *APC* mutation (14). Increasingly, studies have suggested that the position of the APC mutations can determine the level of Wnt activation (8, 15). However, there is little evidence that modulating the Wnt signalling pathway by targeting APC represents a potential therapeutic approach in CRC.

Targeted therapies including cetuximab, which targets the epidermal growth factor receptor (EGFR), are currently approved for treatment of CRC (16). However, cetuximab is ineffective in the presence of *K-Ras* and *B-RAF* mutations that are very common in CRC, therefore indicating that targeting alternative driver mutations may be more effective (17, 18). In drug development, gain-of-function mutations can be targeted by small molecule inhibitors, but where there is loss of a tumor suppressor gene, such as *APC*, it is often very difficult to identify a direct therapeutic target. Moreover, if the tumor suppressor gene has no inherent enzymatic activity to target with a small molecule inhibitor (in the case of APC) this further compounds the issue. To overcome this, the concept of exploiting synthetic lethal relationships for cancer treatment has been proposed as an approach to the targeting of tumor suppressor loss (19, 20). Indeed, we have recently shown that this is a successful approach for the targeting of other non-enzymatic tumor suppressor gene scaffold proteins such as LIMD1 (21). Two genes are said to be synthetically lethal if loss of either gene alone is compatible with viability, but loss of both causes cell death. Therefore, identification of a gene that is, for example synthetically lethal with APC mutation would be clinically relevant. This approach has been exploited successfully in the clinic using inhibitors of poly(ADP-ribose) polymerase (*PARP*) for the treatment of patients with germline mutations in the tumor suppressor genes *BRCA1* and *BRCA2* (22).

With the aim of identifying a tailored therapeutic approach for the treatment of APC-mutant CRC patients, we performed a compound screen to identify drugs that are synthetically lethal with mutant APC. We generated APC-mutant clones using CRISPR-Cas9 editing that express a 75KDa truncated APC protein due to a mutation within the 7^th^ Armadillo repeat that introduces a premature stop codon, where APC interacts with ASEF1/2. The wtAPC and APC-mutant cells were screened with a library of 1170 FDA-approved drugs to identify synthetic lethal compounds. Interestingly, we observed that the APC-mutant cells were extremely sensitive to the statin family of drugs (Lovostatin, Mevastatin & Simvastatin) in comparison to the wtAPC cells. Mechanistic analysis indicated activation of Rac1, followed by a decrease in Wnt signaling, and a decrease in the level of survivin expression, a Wnt target protein, upon statin treatment in the APC-mutant cells only. Significantly, this study elucidates a novel synthetic lethal interaction between APC mutation and statin treatment, which could potentially be exploited for the treatment of a specific subset of APC mutated cancers.

## Materials and Methods

### Cell Culture

The human colorectal cancer RKO cell line was purchased from ATCC and routinely grown in Dulbecco’s Modified Eagles medium (DMEM; Sigma) supplemented with 10% fetal calf serum (FBS; Invitrogen) and 100U/ml penicillin and 100ug/ml streptomycin at 37°C/5% CO_2_. Cell lines were authenticated by STR profiling (DNA Diagnostics Centre Inc.) and routinely mycoplasma tested.

### Generation of APC CRISPR-Cas9 Edited Cells

A non-targeting guide RNA (gRNA) and a gRNA targeting exon 15 within gene APC were individually cloned into the LentiCRISPRv2 vector, according to the manufacturer’s instructions and transduced into RKO cells, which were then selected using puromycin at 0.5 µg/ml followed by clonal selection for gene knockout. APC gRNA sequence used was, 5′-TTGGCATCCTTGTACTTCGC-3′.

### FDA-Approved Compound Library Screen

The FDA-approved compound library incorporating 1170 drugs was purchased from Selleck Chemicals. Cells were plated in 96-well plates and treated with vehicle (0.01% DMSO) or the compound library (average compound concentration of the library in media was 10µM). After 4 days incubation with the drug library, cell viability was assessed using the CellTiter Glo assay (Promega) according to the manufacturer’s instructions. Luminescence readings from each well were log transformed and normalized according to the median signal on each plate and then standardized by use of a Z-score statistic, using the median absolute deviation to estimate the variation in each screen. Z-scores were compared to identify compounds that cause selective loss of viability in APC^mut^*2 cells, in comparison to Control*1 cells. For validation experiments, cells were treated with increasing concentrations of Lovastatin, Mevastatin and Simvastatin and cell viability was assayed after 5 days. Lovastatin, Mevastatin, Simvastatin, Mevalonic acid and EHT1864 were purchased from Sigma-Aldrich. GGTTI-298 was purchased from Calbiochem. FTI-277 was purchased from Santa Cruz.

### Cell Viability Assays

Cells were seeded in 96-well plates (1000 cells/well) 24h before treatment. Lovastatin, Mevastatin and Simvastatin were serially diluted in DMEM media, as indicated. After 4 days, cell viability was assessed with the ATP-based, luminescence assay, CellTiter-Glo (Promega).

### TCF/LEF Reporter Assay

To measure TCF/LEF, we used plasmid-based TCF/LEF luciferase reporter vectors (Cignal Report; Qiagen), according to the manufacturer’s instructions. This assay includes the inducible transcription factor-responsive construct expressing firefly luciferase, and the constitutively expressing Renilla luciferase construct which acts as an internal control. Cells were transfected with 1 μL TCF/LEF reporter plasmid and 0.25 μL FuGENE in Opti-MEM (Invitrogen), and seeded into individual wells of 96-well opaque plates. After 5 days, the Dual-Luciferase Reporter Assay System (Promega) was used to develop luciferase signals, which were measured using a Perkin Elmer 1420 multilabel counter victor 3 plate reader. Firefly luciferase levels were normalized to renilla luciferase.

### Western Blotting

Cells were lysed in 20 mM Tris (pH 8), 200 mM NaCl, 1 mM EDTA, 0.5% (v/v) NP40, 10% glycerol, supplemented with protease inhibitors (Roche). Equivalent amounts of protein were electrophoresed on either 3-8% tris-acetate or 4-12% tris-glycine Novex precast gels (Invitrogen) and transferred to nitrocellulose membrane. After blocking for 1h in 1xTBS/5% non-fat dried milk, membranes were incubated overnight at 4°C in primary antibody (**Table 1**). Membranes were incubated with anti-IgG-horseradish peroxidase and visualized by chemiluminescent detection (Supersignal West Pico Chemiluminescent Substrate, Pierce). Immunoblotting for β-actin or β-tubulin were performed as loading controls.

**Table 1:** Antibodies.

Protein	Catalog #	Source	Antibody dilution	Gel used
APC	#2504	Cell signaling	1:250	3-8% tris-acetate
β-catenin	#4270	Cell signaling	1:5000	4-12% tris-glycine
unphosphorylated ser33/ser37/thr41				
β-catenin	ab32572	Abcam	1:10,000	4-12% tris-glycine
Pak1	#2602	Cell signaling	1:1000	4-12% tris-glycine
Pak1	#2606	Cell signaling	1:1000	4-12% tris-glycine
phosphorylated ser144				
Rac1	#16118	ThermoScientific	1:1000	4-12% tris-glycine
Rho	#16116	ThermoScientific	1:650	4-12% tris-glycine
Survivin	ab76424	Abcam	1:1000	4-12% tris-glycine
β-tubulin	T8328	Sigma	1:10,000	4-12% tris-glycine
β-actin-HRP	#5125	Cell signaling	1:10,000	4-12% tris-glycine

### siRNA Transfections

For siRNA transfections, cells were transfected with individual siRNA oligos (**Table 2**; Dharmacon) using Lipofectamine RNAiMax or Lipofectamine 2000 (Invitrogen) according to the manufacturer’s instructions. As a control for each experiment, cells were left un-transfected or transfected with a non-targeting control siRNA and concurrently analyzed.

**Table 2:** siRNA.

siRNA	Catalog #	Source
BIRC5 (Survivin)	M-003459-03	Dharmacon
Non-targeting Ctrl	D-001206-14	Dharmacon

### Rac1 and Rho Activation Assays

Rac1 activity was assessed using the Active Rac1 Pulldown and Detection kit (16118, Thermo Fisher Scientific) and Rho activity was assessed using the Active Rho Pulldown and Detection kit (16116, Thermo Fisher Scientific) in accordance with the manufacturer’s instructions. Briefly, RKO Control*1 and *2 and APC^mut^ *1 and *2 lysates (500mg) were centrifuged at 16,000 *g* at 4°C for 15 min, and then the supernatants were transferred to a new tube. Rac1-activation controls, either GTPyS (10 mM) or GDP (100 mM) were added and incubated at 30°C for 15 min in a shaker. The mixtures were then incubated with glutathione resin beads and glutathione S-transferase-fused fusion protein (Rac1-binding domain of Pak1 or Rho-binding domain of Rhotekin) at 4°C. After 1 hr, the beads and proteins bound to the fusion protein were washed three times with wash buffer at 4°C, eluted in SDS sample buffer, and analyzed for bound Rac1 or Rho by western blotting using anti-Rac1 or anti-Rho antibody, respectively.

### Immunofluorescence

Cells were seeded on poly-L-lysine coated coverslips. After permeablisation with 0.1% triton and fixation with 3.7% paraformaldehyde, cells were incubated in anti- β-catenin antibody (#ab32572, Abcam) in 2% BSA for 40 minutes at 37°C. Cells were incubated in secondary antibody in 2% BSA for 30 minutes at 37°C and stained with DAPI for nuclei staining. Coverslips were mounted and imaged with LSM 710. At least 150 cells were counted per condition. Images were analyzed and quantified using ImageJ software.

### Statistical Analysis

Unless stated otherwise, data represent standard error of the mean of at least three independent experiments. The two-tailed paired Student’s t test was used to determine statistical significant with p<0.05 regarded as significant.

## Results

### APC Mutation Confers Sensitivity to Statin Treatment

**Figure 1:**
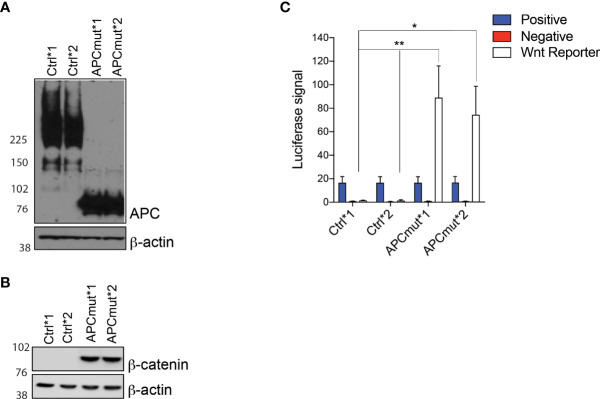
APC mutation is associated with increased Wnt signaling **(A)** Western blot analysis of protein lysates from RKO Control*1, Control*2, APCmut*1 and APCmut*2 cells. Protein was extracted and expression was analyzed using APC and β-actin antibodies. β-actin was used as a loading control. **(B)** Western blot analysis of protein lysates from RKO Control*1, Control*2, APCmut*1 and APCmut*2 cells. Protein was extracted and expression was analyzed using total β-catenin and β-actin antibodies. β-actin was used as a loading control. **(C)** APC mutation increased TCF/LEF activity. RKO Control*1, Control*2, APCmut*1 and APCmut*2 cells were transfected with TCF/LEF luciferase reporter vectors and luciferase signals were analyzed using the Dual-Luciferase Reporter Assay System. Firefly luciferase levels were normalized to renilla luciferase levels. Positive and negative controls were included. Data represent mean ± SEM of three independent experiments. **p ≤ 0.005; *p ≤ 0.05.

With the aim of identifying a tailored therapeutic approach for the treatment of a large proportion of CRC patients, we performed a compound screen to identify drugs that are synthetically lethal with mutant APC. To this end, we generated APC-mutated CRC cells using CRISPR-Cas9 in the APC wild-type (wt) RKO CRC cells. We established two CRISPR-Cas9 non-targeting gRNA control cell lines (Control *1 and *2) and two CRISPR-Cas9 APC-mutant clones (APC^mut^ *1 and *2). The APC-mutant clones express a 75KDa truncated APC protein and activate Wnt (**Figures 1A–C**) due to a mutation in the 7^th^ Armadillo repeat where APC interacts with ASEF1/2.

**Figure 2:**
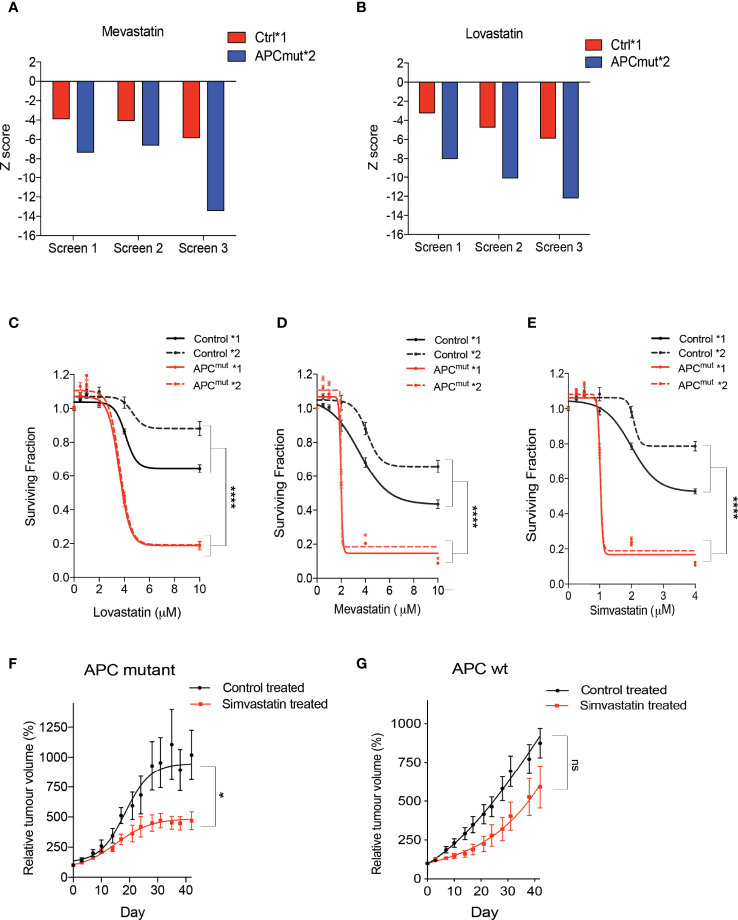
Statin compounds are synthetically lethal in APC mutated cells, *in vitro* and *in vivo.* RKO Control*1, and APCmut*2 cells were plated in 96-well plates and treated with vehicle (0.01% DMSO) or the compound library (average compound concentration of the library in media was 10µM). After 4 days incubation with the drug library, cell viability was assessed. Z-scores were compared to identify compounds that cause selective loss of viability in APC mutated cells, in comparison to wt APC cells. Graphs represent Z-scores for **(A)** mevastatin and **(B)** lovastatin for each of the three replicate drug screens. RKO Control*1, Control*2, APCmut*1 and APCmut*2 cells were treated with increasing concentrations of **(C)** lovastatin (0, 2 µM, 4 µM, 6 µM, 8 µM & 10 µM), **(D)** mevastatin (0, 2 µM, 4 µM, 6 µM, 8 µM & 10 µM) and **(E)** simvastatin (0, 1 µM, 2 µM, 3 µM & 4 µM). After 4 days treatment, cell viability was measured using an ATP-based luminescence assay. *In vivo* efficacy experiments were performed on 40 NMRI nu/nu mice injected with either APC mutant patient derived tumor cells **(F)** or wt APC patient derived tumor cells **(G)**. When the tumors were measurable, mice were treated daily by gavage with 50mg/kg simvastatin or vehicle. Tumors were measured twice a week and tumor size was normalized to initial treatment measurements. **(C–E)** Data represent mean ± SEM of three independent experiments. *p ≤ 0.05; ****p ≤ 0.00005; ns, non-significant.

We screened the two RKO wtAPC control cell lines and both the RKO APC-mutated cell clones, with a library of 1170 FDA-approved drugs to identify synthetic lethal compounds (**Supplementary Figure 1**). Interestingly, analysis of our screen revealed that the RKO APC-mutated cells were extremely sensitive to the statin group of drugs (Lovastatin & Mevastatin; **Figures 2A, B*****)***. To determine whether this was an effect due to the compound screening approach and/or the specific concentration used, we next treated the RKO Control *1 and *2 and APC^mut^ *1 and *2 cell lines with increasing concentrations of three different statin compounds (Lovastatin, Mevastatin & Simvastatin; **Figures 2C–E**). We observed a significant difference in sensitivity of the APC-mutated cells, in comparison to the RKO Control cells, with SF50’s in the low μM range (between 1-4 μM). Next, to ensure the synthetic lethality observed between the APC mutation and statin treatment, was not an artefact of our *in vitro* cell culture models, an *in vivo* CRC cancer model was used that represented APC-mutant patient tumors. To this end, we investigated sensitivity to statin treatment in a patient-derived xenograft *in vivo* model (PDX) of APC mutated CRC. The wtAPC and APC-mutated primary patient tumor cells were successfully engrafted into nude mice and once established were treated with simvastatin. (**Figures 2F, G*****)***. Treatment with simvastatin was found to slow down the growth of the PDX APC-mutated tumors, but not wtAPC tumors. Taken together, these data indicate that APC-mutant CRC tumor cells are differentially and exquisitely sensitive to treatment with statin compounds, both *in vitro* and *in vivo*.

### Sensitivity to Statins in APC-Mutant Cells Is Mediated *via* Geranylgeranyl Pyrophosphate Protein Prenylation

Statins are small molecule inhibitors of 3-Hydroxy-3-Methylglutaryl-CoA Reductase (HMGCR) in the mevalonate pathway, approved for the prevention of cardiovascular disease. HMGCR catalyses the conversion of 3-hydroxyl-3-methylglutaryl coenzyme A (HMG-CoA) to mevalonate and this is the rate limiting step in the pathway involved in cholesterol production (23). Inhibition of HMGCR results in the reduction of plasma cholesterol levels.

**Figure 3:**
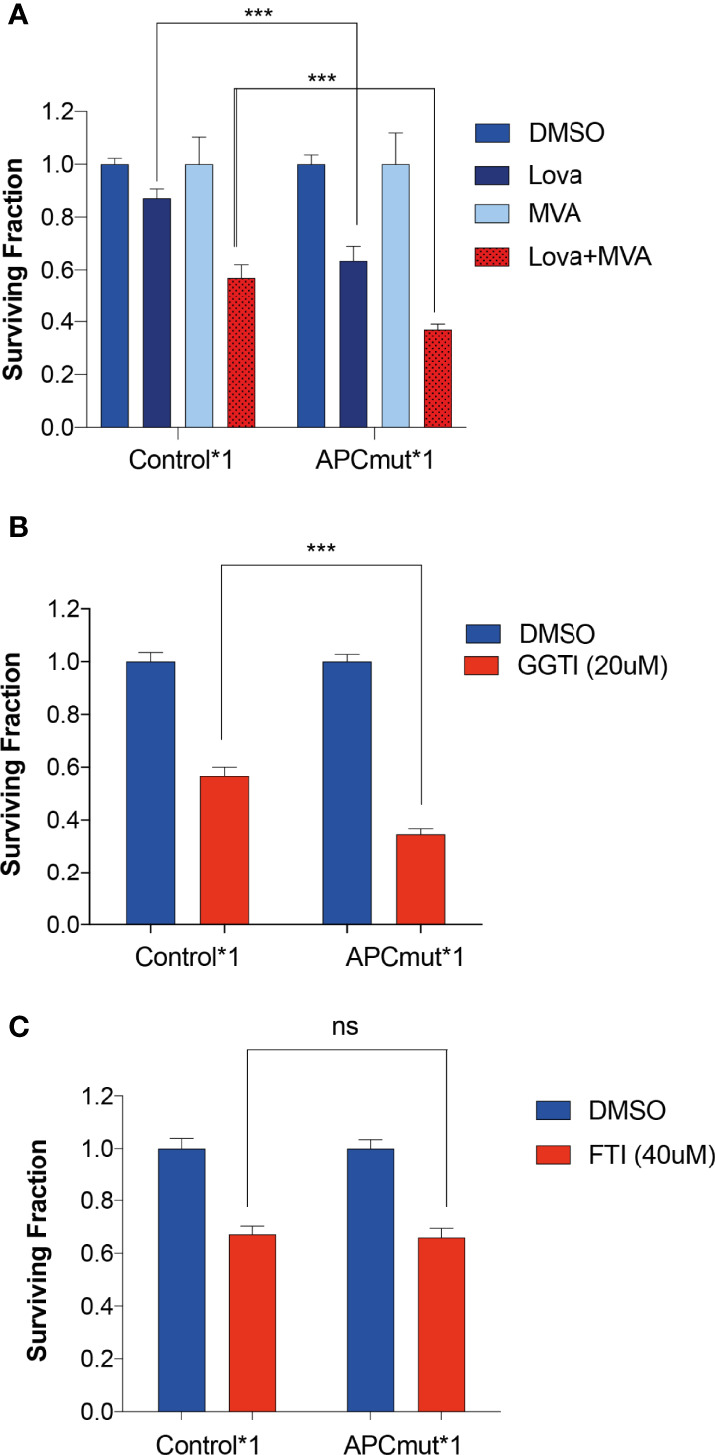
Sensitivity to statins in APC-mutant cells is mediated *via* the Melavonate pathway and geranylgeranyl pyrophosphate protein prenylation. **(A)** RKO Control*1, and APCmut*1 cells were pre-treated with either vehicle (0.01% DMSO) or MVA (100μM) for 1 hr, followed by lovastatin (6μM) treatment, as indicated. After 48 hrs, cell viability was assessed using an ATP-based luminescence assay. **(B)** RKO Control*1 and APCmut*1 cells were with either vehicle (0.01% DMSO) or GGTI (20μM). After 48 hts, cell viability was assessed using an ATP-based luminescence assay. **(C)** RKO Control*1 and APCmut*1 cells were treated with either vehicle (0.01% DMSO) or FTI (40μM). After 48 hrs, cell viability was assessed using an ATP-based luminescence assay. **(A–C)** Data represent mean ± SEM of three independent experiments. ***p ≤ 0.0005; ns, non-significant.

Previous studies have shown that HMGCR catalyses the conversion of HMG-CoA into Mevalonate [also known as Mevalonic acid (MVA)]. Therefore, to understand the role of the mevalonate pathway in the sensitivity of APC-mutated cells to statins, we next investigated whether addition of MVA could rescue the effect of statin treatment in our cell lines. RKO Control*1 and APC^mut^*1 cells were pre-treated with MVA for 1hr, followed by the addition of lovastatin for 48 hours (**Figure 3A**). Unexpectedly, instead of rescuing the sensitivity, we observed a significantly greater decrease in cell viability in the APC^mut^*1 cells when treated with MVA, followed by lovastatin treatment. This effect was dependent on lovastatin treatment, as cells treated with MVA alone remained viable.

Given the differential sensitivity to MVA upon lovastatin treatment in the APC mutated cells, we investigated this effect further by interrogating downstream of the mevalonate pathway. Inhibition of HMGCR *via* statin treatment has been shown to be involved in the reduction in isoprenoids including farnesyl pyrophosphate (FPP) and geranylgeranyl pyrophosphate (GGPP). Isoprenoids are added to specific proteins as a post translational modification, known as prenylation. Prenylation adds long hydrophobic molecules to proteins to enable them to anchor to cell membranes. Prenylation is mediated by either farnesyl transferase (FTase) or geranylgeranyl transferase (GGTase). To investigate the role of isoprenoids in statin sensitivity, we analyzed whether inhibiting GGTase or FTase with a geranylgeranyltransferase inhibitor (GGTI-298) or a farnesyltransferase inhibitor (FTI-277) respectively, would also induce sensitivity in the APC-mutated cells. GGTI-298 prevents the formation of GGPP prenylated proteins and FTI-277 prevents the formation of FPP prenylated proteins. To this end, we treated the RKO Control*1 and APC^mut^*1 cells with GGTI-298 or FTI-277 and measured cell viability (**Figures 3B, C****)**. Interestingly, we observed that the APC^mut^*1 cells were significantly more sensitive to GGTI compared to the RKO Control*1 cells. In comparison, treatment with FTI-277 showed no differential sensitivity in the RKO Control*1 and APC^mut^*1 cells. This suggests that statins may mediate their selective sensitivity in APC^mut^ cells through proteins which undergo GGPP prenylation.

### Active Rac1 Levels Increase Upon Statin Treatment in APC-Mutated Cells

Our data suggest that proteins which undergo GGPP prenylation could have a role in the mechanism of sensitivity to statins in APC mutated cells. Protein families which undergo prenylation include Rac1, Rho and Cdc42 (24). To investigate this further, we first analyzed the GGPP prenylated protein, Rac1 because it has previously been shown to be important in the Wnt signaling pathway either by transporting β-catenin into the nucleus or by promoting the formation of β-catenin-TCF/LEF complexes (25, 26).

**Figure 4:**
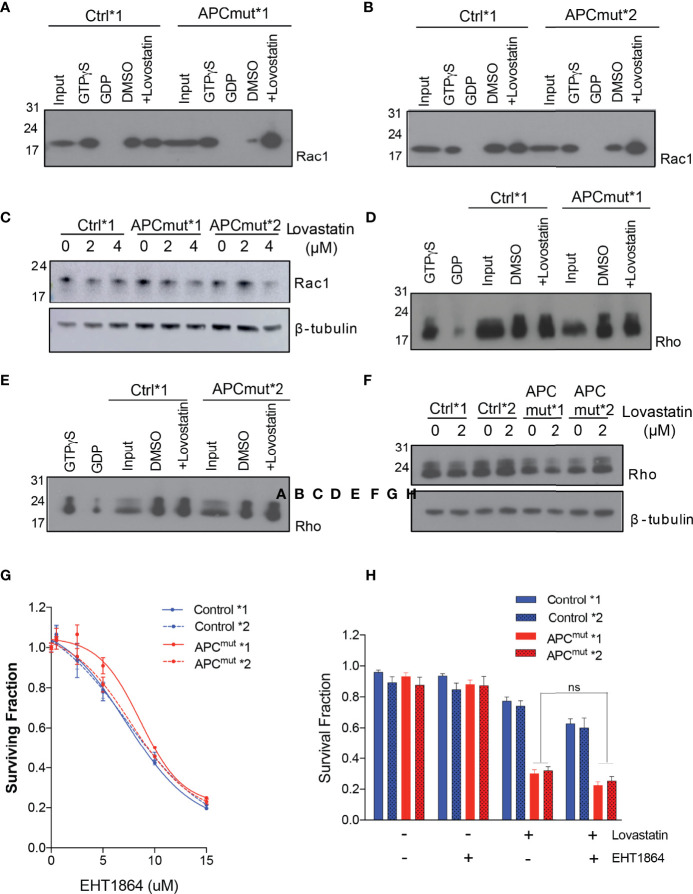
Statin treatment increases active Rac1 levels in APC-mutant cells. **(A)** RKO Control*1 and APCmut*1 and **(B)** RKO Control*2 and APCmut*2 cells were treated with either vehicle (0.01% DMSO) or 2 μM lovastatin for 72 hours. Whole cell lysates were collected and incubated with a column containing a resin with a GST-fusion protein with the Rac1-binding domain of Pak1. Upon immunoprecipitation, samples were immunoblotted for Rac1 expression. The input lane shows levels of total Rac1. GTPyS and GDP treated samples were included as positive and negative controls for Rac1 activity, respectively. **(C)** RKO Control*1, Control*2, APCmut*1 and APCmut*2 cells treated with either vehicle (0.01% DMSO), 2 μM or 4 μM lovastatin for 72 hours. Protein was extracted and samples were immunoblotted for total Rac1 expression. β-tubulin was used as a loading control. **(D)** RKO Control*1 and APCmut*1 and **(E)** RKO Control*2 and APCmut*2 cells were treated with either vehicle (0.01% DMSO) or 2 μM lovastatin for 72 hours. Whole cell lysates were collected and incubated with a column containing a resin with the GST-fusion protein containing the Rho-binding domain of Rhotekin. Upon immunoprecipitation, samples were immunoblotted for Rho expression. The input lane shows levels of total Rho. GTPyS and GDP treated samples were included as positive and negative controls for Rho activity, respectively. **(F)** RKO Control*1, Control*2, APCmut*1 and APCmut*2 cells treated with either vehicle (0.01% DMSO), 2 μM or 4 μM lovastatin for 72 hours. Protein was extracted and samples were immunoblotted for total Rho expression. β-tubulin was used as a loading control. **(G)** RKO Control*1, Control*2, APCmut*1 and APCmut*2 cells were treated with either vehicle (0.01% DMSO) or increasing concentrations of the Rac1 inhibitor, EHT1864 (0, 5 µM, 10 µM, 15 µM). After 4 days treatment, cell viability was measured using an ATP-based luminescence assay. **(H)** RKO Control*1, Control*2, APCmut*1 and APCmut*2 cells were treated with either vehicle (0.01% DMSO), lovastatin (6 µM) or EHT1864, alone or in combination. After 96 hrs treatment, cell viability was measured using an ATP-based luminescence assay. ns, non-significant.

To analyse Rac1 activity, we treated the RKO Control*1 and *2 and APC^mut^*1 and *2 cells with and without lovastatin for 72 hours and collected whole cell lysates. As a control for Rac1 activation, the parental RKO wtAPC cell line was treated with either GDP (inactivates Rac1) or GTPyS (activates Rac1). Protein lysates were run through a column containing a resin with a GST-fusion protein with the Rac1-binding domain of Pak1, where only active Rac1 interacts with Pak1 (27). Upon immunoprecipitation, lysates were then analyzed by western blotting for Rac1 expression (**Figure 4*****).*** We observed that the RKO Control*1 lysates had a similar level of active Rac1, before and after lovastatin treatment (**Figures 4A, B*****)***. Whereas the APC^mut^*1 and *2 cell lysates had a lower basal level of active Rac1, compared to the control cells. However, upon lovastatin treatment, the level of active Rac1 was greatly increased. To determine whether this was due to different levels of total Rac1 or active Rac1 specifically, we treated RKO Control*1 and *2 and APC^mut^*1 and *2 cells with lovastatin and after 72 hours, lysates were immunoblotted and probed for total Rac1 expression (**Figure 4C**). Interestingly, total Rac1 expression did not increase in the APC^mut^ cells after treatment, suggesting that Rac1 activity was specifically increased in the APC mutant cells upon lovastatin treatment.

To understand whether increased Rac1 activity in APCmut cells upon lovastatin treatment was specific to Rac1 or whether statin treatment would increase the activity of other proteins which undergo GGPP prenylation, we next analyzed levels of the GGPP prenylated protein Rho. To analyse Rho activity, we treated the RKO Control*1 and *2 and APCmut*1 and *2 cells with and without lovastatin for 72 hours and collected whole cell lysates. Protein lysates were run through a column containing a resin with the GST-fusion protein containing the Rho-binding domain of Rhotekin, which binds active Rho only. Upon immunoprecipitation, lysates were immunoblotted and probed for Rho expression. In contrast to Rac1, we detected no change in the level of active Rho upon lovastatin treatment in the RKO Control*1 and *2 and APCmut*1 and *2 cells (**Figures 4D, E**). We also analyzed total Rho expression in whole cell lysates from RKO Control*1 and *2 and APCmut*1 and *2 treated with lovastatin for 72 hours (**Figure 4F**). The expression of total Rho did not change upon statin treatment in the APC wt or APC mutant cell lines. Therefore, the increased activity observed upon lovastatin treatment in APCmut cells was specific to Rac1.

As we observed a significant activation of Rac1 upon statin treatment in the APC^mut^ cell lines, we next investigated whether Rac1 activation was causing the sensitivity of the APC^mut^ cells to statin treatment. Firstly, we treated our cells with the Rac1 inhibitor EHT1864, which prevents Rac1 binding to GTP and therefore prevents its activation [(28) **Figure 4G**]. Interestingly, we did not observe any differential sensitivity to Rac1 inhibition in the RKO Control*1 and *2 and APC^mut^*1 and *2 cells, suggesting that increased Rac1 activity was not mediating the differential sensitivity to statin treatment in our APC-mutant cells. We next investigated if inhibiting Rac1 activation in combination with statin treatment would rescue the sensitivity of the APC mutant cell lines to statin treatment. To this end, the RKO Control*1 and *2 and APC^mut^*1 and *2 cells were treated with either EHT1864 or lovastatin alone or in combination and cell viability was analyzed (**Figure 4H**). Our data showed that addition of EHT1864 did not rescue the sensitivity of the APC mutant cell lines to lovastatin. This suggests that the increase in Rac1 activation was not solely responsible for the reduced cell viability in the APC mutant cell lines, upon statin treatment.

### Statin Treatment Increases β-Catenin Transport Into the Nucleus in APC-Mutated Cells

Rac1 has previously been shown to play a role in the Wnt signalling pathway. Active Rac1 has been shown to activate JNK2, which then phosphorylates β-catenin at ser191 and ser605, enabling β-catenin to translocate to the nucleus (25, 26). Given our data showed an increase in active Rac1 following lovastatin treatment in our APC-mutated cells, we investigated whether statin treatment would influence the ability of β-catenin to translocate from the cytoplasm to the nucleus, in APC-mutant cells, due to Rac1.

**Figure 5:**
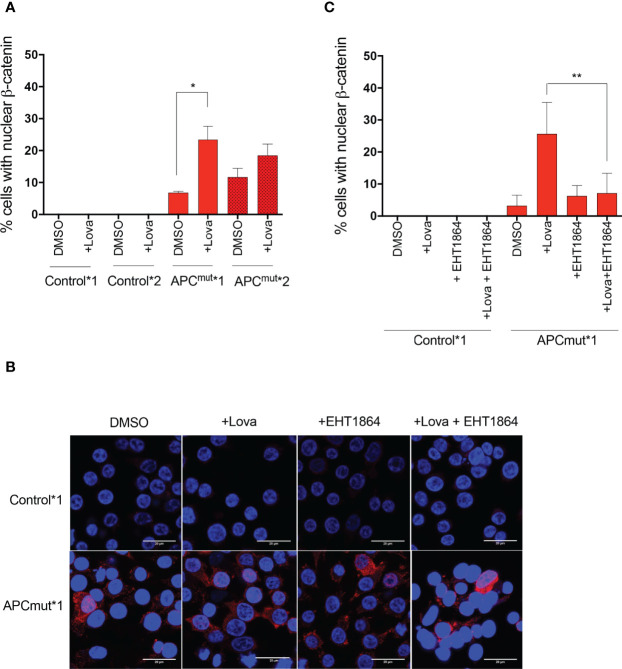
Statin treatment increases β-catenin transport into the nucleus *via* Rac1 in APC-mutated cells. **(A)** RKO Control*1, Control*2, APCmut*1 and APCmut*2 cells were treated with either vehicle (0.01% DMSO) or 4 μM lovastatin for 72 hours before fixing. Cells were then incubated with anti-β-catenin antibody and visualized *via* confocal microscopy. Total number of cells with nuclear-localization of β-catenin were quantified and expressed as a % over total number of DAPI-stained cells. **(B)** Representative images shown of RKO Control*1 and APCmut*1 cells treated with either vehicle (0.01% DMSO), 4 μM lovastatin or 1 μM EHT1864, alone or in combination for 72 hours. DAPI staining is in blue, total β-catenin is in red. Scale bar indicates 20 μM. **(C)** RKO Control*1 and APCmut*1 cells were treated with either vehicle (0.01% DMSO), 4 μM lovastatin or 1 μM EHT1864, alone or in combination for 72 hours before fixing. Cells were then incubated with anti-β-catenin antibody and visualized *via* confocal microscopy. Total number of cells with nuclear-localization of β-catenin were quantified and expressed as a % over total number of DAPI-stained cells. *p = 0.05, **p ≤0.005.

To investigate this, the RKO Control*1 and *2 and APC^mut^*1 and *2 cell lines were treated with either vehicle or lovastatin for 72 hours. The cells were then fixed and stained with DAPI and total β-catenin antibody (**Figures 5A; B**, **Supplementary Figure 2A**). Interestingly, in the APC^mut^*1 cells, we observed a significant increase in cells with nuclear β-catenin following lovastatin treatment, suggesting increased β-catenin binding to TCF/LEF and activation of Wnt target genes (**Figure 5A**). Statin treatment did not increase β-catenin expression or localization in the Control*1 and *2 cells.

To determine whether increased Rac1 activity was required for the localization of β-catenin in APC-mutated cells following statin treatment, we inhibited Rac1 using EHT1864 in combination with lovastatin treatment in the RKO Control*1 and *2 and APC^mut^*1 and *2 cells and analyzed levels of total β-catenin in the cytoplasm and nucleus (**Figures 5B, C*****;*** **Supplementary Figure 2B**). In the APC^mut^*1 and *2 cells, we previously observed decreased cytoplasmic β-catenin and increased nuclear β-catenin upon lovastatin treatment alone (**Figure 5C*****;*** **Supplementary Figure 2B**). However, upon treatment of the APC^mut^*1 and *2 cells with both lovastatin and EHT1864 combined, nuclear β-catenin levels remained similar to the vehicle-treated cells (**Figure 5C*****;*** **Supplementary Figure 2B**). Overall, this data suggests that an increase in Rac1 activity upon statin treatment was required for the localization of β-catenin from the cytoplasm into the nucleus of APC^mut^*1 and *2 cells.

### Statin Treatment Is Associated With Reduced Pak1 Phosphorylation, Leading to Reduced Expression of the Wnt Target Gene, Survivin in APC-Mutated Cells

Given that we observed increased nuclear β-catenin upon statin treatment in the APC^mut^*1 and *2 cells, we investigated whether this resulted in an increase in Wnt signaling and expression of Wnt target genes. Firstly, we analyzed protein levels of total β-catenin and unphosphorylated β-catenin after statin treatment. When the Wnt pathway is inactive, β-catenin is phosphorylated at ser33, ser37 and thr41 and signals β-catenin for degradation, resulting in significantly reduced expression levels. When the pathway is active, β-catenin remains unphosphorylated, enabling β-catenin to accumulate in the nucleus and activate Wnt target genes.

**Figure 6:**
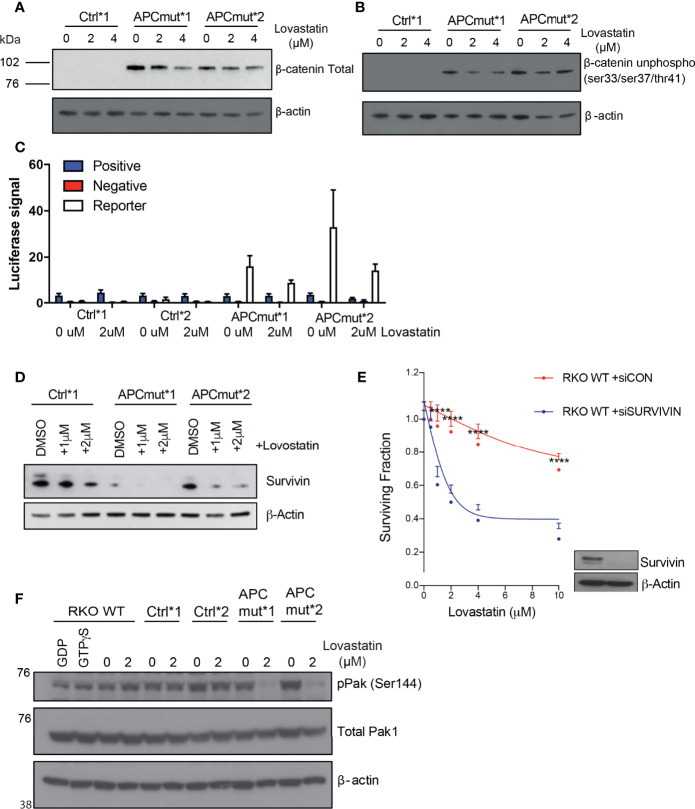
APC mutant cells have reduced survivin expression which mediates sensitivity to statin treatment. **(A)** RKO Control*1, APCmut*1 and APCmut*2 cells were treated with either vehicle (0.01% DMSO), 2 μM or 4 μM lovastatin for 72 hours. Protein was extracted and samples were immunoblotted for total β-catenin expression. β-actin was used as a loading control. **(B)** RKO Control*1, APCmut*1 and APCmut*2 cells were treated with either vehicle (0.01% DMSO), 2 μM or 4 μM lovastatin for 72 hours. Protein was extracted and samples were immunoblotted for unphosphorylated β-catenin (ser33/ser37/thr41) expression. β-actin was used as a loading control. **(C)** RKO Control*1, Control*2, APCmut*1 and APCmut*2 cells were treated with either vehicle (0.01% DMSO) or 2 μM lovastatin for 72 hours, followed by transfection with TCF/LEF luciferase reporter vectors. Luciferase signals were analyzed using the Dual-Luciferase Reporter Assay System. Firefly luciferase levels were normalized to renilla luciferase levels. Positive and negative controls were included. **(D)** RKO Control*1, APCmut*1 and APCmut*2 cells were treated with either vehicle (0.01% DMSO), 1 μM or 2 μM lovastatin for 72 hours. Protein was extracted and samples were immunoblotted for survivin expression. β-actin was used as a loading control. **(E)** RKO WT APC cells were transfected with either non-targeting siRNA (siCON) or siRNA targeting survivin (siSurvivin). After 24 hrs, cells were treated with either vehicle (0.01% DMSO) or increasing concentrations (2 µM, 4 µM, 6 µM, 8 µM, 10 µM) of lovastatin for 72 hours. Cell viability was assessed using an ATP-based luminescence assay. ****p ≤ 0.00005. **(F)** RKO WT APC, Control*1, Control*2, APCmut*1 and APCmut*2 cells were treated with either vehicle (0.01% DMSO) or 2 μM lovastatin for 72 hours. Protein was extracted and samples were immunoblotted for phosphorylated Pak1/2/3 and total Pak1 expression. β-actin was used as a loading control.

To investigate protein expression levels of total and phosphorylated β-catenin, we treated the RKO Control*1 and *2 and APC^mut^*1 and *2 cells with vehicle or lovastatin and immunoblotted whole cell lysates. We observed decreased expression of both total and unphosphorylated β-catenin in the APC^mut^*1 and *2 cells after statin treatment suggesting decreased activation of Wnt signaling (**Figures 6A, B*****;*** **Supplementary Figure 2C**). To further our analysis of the Wnt signalling pathway, we performed a TCF/LEF luciferase assay, which measures the level of TCF/LEF binding to TRE and is an indicator of the activation of the Wnt signalling pathway. In keeping with the decreased expression of unphosphorylated and total β-catenin, treatment with lovastatin caused a decrease in Wnt activation in the RKO APC^mut^ *1 and *2 cells. (**Figure 6C**). This decrease in total and unphosphorylated (active) β-catenin expression and decreased Wnt activation using the TCF/LEF reporter assay after statin treatment in APC-mutated cells was surprising, as we had previously observed an increase in nuclear β-catenin upon statin treatment in APC-mutated cells. Our data therefore suggested that statins may cause β-catenin to accumulate in the nucleus, but in this case β-catenin was unable to active Wnt signalling.

To investigate this further, we analyzed the expression of the Wnt-target gene survivin, as statin treatment can induce decreased expression of this anti-apoptotic protein in CRC (29, 30). Therefore, we investigated whether increased nuclear β-catenin upon statin treatment in APC-mutated cells, would result in increased survivin expression. RKO Control*1 and APC^mut^ *1 and *2 cells were treated with either vehicle or lovastatin and lysates were analyzed by western blot analysis. After 72 hours of statin treatment, we observed that statin treatment caused a significantly greater decrease in survivin expression in the APC^mut^ *1 and *2 cell lines, compared to the Control*1 cells (**Figure 6D**). To understand whether survivin levels were driving the sensitivity of our cells to statins, we depleted survivin in the wtAPC RKO cell line using siRNA and measured cell viability following statin treatment (**Figure 6E**). Upon depletion of survivin, cells were significantly more sensitive to lovastatin, in comparison to the siControl (siCON) transfected RKO cells. Therefore, our data suggests that reduced survivin expression can influence the sensitivity of CRC cells to lovastatin treatment.

Previous studies have shown that the serine threonine kinase downstream of Rac1, Pak1 can phosphorylate β-catenin at ser675, resulting in β-catenin stabilization and enhanced transcription of Wnt target genes (31). Pak1 phosphorylation at ser144 is unique to Rac1 and Cdc42, and results in Pak1 activation (32). To investigate Pak1 phosphorylation, RKO wt, Control*1 and *2 and APC^mut^ *1 and *2 cell lines were treated with either vehicle or lovastatin for 72 hours. Following treatment, protein lysates were immunoblotted and probed for phosphorylated Pak1 (ser144) and total Pak1 expression (**Figure 6F*****).*** Upon statin treatment, there was a significant decrease in phosphorylated Pak1 expression in the APC mutant cells only. No difference was observed in the wtAPC control lines following treatment. Therefore, these exciting data indicate that statin treatment can reduce the activation of Pak1 in APC-mutant cells.

Taken together, our data has defined a novel synthetic lethal interaction upon APC loss and statin treatment. We have elucidated for the first time that APC-mutated CRC cells are differentially sensitive to statin treatment, due to an increase in Rac1 activity which induced translocation of β-catenin to the nucleus. β-catenin accumulates in the nucleus but is prevented from activating Wnt target genes due to the inhibition of Pak1 phosphorylation resulting in reduced transcription of Wnt target genes, such as survivin. It is this reduced expression of survivin expression that results in the synthetic lethal interaction upon statin treatment in APC-mutated cells therefore defining this new pathway as a potential new therapeutic target in CRC.

## Discussion

Although APC was identified as a key genetic driver of CRC more than 30 years ago, this knowledge has not yet been translated effectively into the clinic and successful tailored therapy for CRC patients is still lacking. Despite the overwhelming evidence that Wnt signaling drives CRC, targeted Wnt therapies have not been successful in the clinic. Many studies searching for synthetic lethal relationships in CRC have focused on looking for relationships with other major mutations such as KRAS (33–35). Previous studies have identified specific synthetic lethal relationships with APC mutations. One group identified that APC mutant CRC was synthetically lethal with NSAIDs (36). Mutant APC increased levels of the Wnt target gene c-Myc, resulting in higher B3 interacting-domain death agonist (BID) activation and NSAIDs further activated BID, resulting in APC mutant cell specific death, leaving the normal wtAPC cells unharmed (36). Also, NSAIDs have been reported to inhibit COX2 which is thought to be synthetically lethal with APC mutations because APC mutant cells show increased expression of COX2 (37, 38). Additionally, there is potential to target Tankyrase (TNKS) and proteins upstream of TNKS to selectively kill APC mutant cells. TNKS destabilises Axin which is the rate limiting component of the β-catenin, therefore the inhibition of TNKS increases Axin and levels of the β-catenin destruction complex, resulting in the inhibition of Wnt signalling (39). Interestingly, the length and position of the APC mutation has been linked to the sensitivity to TNKS inhibitors, cell lines lacking all seven 20aa repeats were more sensitive than those with two or more 20aa repeats (40). Unfortunately, TNKS inhibitors are often toxic to normal intestinal cells. However a study has identified that PrxII regulates TNKS only in APC mutant CRC and the inhibition of PrxII results in APC mutant cell specific death (41, 42). Further studies have identified the compound TASIN as a potential therapeutic to treat APC mutant cells (43, 44). TASIN-1 inhibits a component of the cholesterol synthesis pathway and it is thought that APC mutant cells are defective in responding to decreases in cholesterol, resulting in APC mutant specific cell death. This indicates the cholesterol synthesis pathway, beyond statin treatment, could be an ideal target for the treatment of APC mutant CRC.

Our data suggests that upon statin treatment levels of survivin decrease to a greater extent in the APC mutant lines compared to the APC wildtype lines, suggesting that in the APC mutant lines the level of survivin decreases below a threshold tolerated, resulting in the induction of apoptosis. This is supported by other studies in CRC cells showing that statin treatment induced a decrease in survivin levels (30, 45). Interestingly in different cancer types including lung, hepatocellular carcinoma and head and neck squamous cell carcinoma, the downregulation of survivin was also considered to be part of the mechanism of action of statins (46–48). Silencing survivin can increase sensitivity to statin treatment in wtAPC expressing cells, therefore suggesting survivin levels play a key role in the response to statins in CRC.

Many studies have investigated the potential of statins in chemoprevention. Significantly, one preclinical study was suggested that combination therapy of atorvastatin together with low doses of the NSAID, celecoxib, can significantly increase the chemopreventive efficacy in an APC^Min^/+ mice (49). Case control studies have shown varying protective effects; for example, one study identified statin treatment resulted in a 4% decreased risk of CRC, whilst other studies have shown no protective effect (50, 51). Unfortunately, randomised studies have not supported a protective effect (51). The studies used to investigate the association between statins and CRC have been designed to test the safety of statins to treat cardiovascular disease and are, therefore, not designed to assess cancer risk, as the follow-up period is often too short. Additionally, cardiovascular disease patients are at higher risk of CRC because risk factors associated with cardiovascular disease also include CRC risk factors such as low physical activity and poor diet (51). Interestingly, a large meta-analysis of 42 studies which included case-control studies, cohort studies and randomised control trials, concluded that statins have a slight protective effect, but that long-term use does not seem to influence CRC risk (52). It has also been suggested that the link between statins and reduced cancer risk may be due to cholesterol levels associated with those taking statins, rather than the result of statin treatment *per se* (53).

Our data suggests that regulation of survivin levels, following statin treatment in APC-mutant cells may be the key to fully understanding the link between statins and CRC.

## Data Availability Statement

The datasets presented in this article are not readily available. Requests to access the datasets should be directed to sarah.martin@qmul.ac.uk.

## Ethics Statement

The animal study was reviewed and approved by Charles Rivers.

## Author Contributions

Experimental design and execution was conducted by HS, WT, MF, AS, and SM. Data interpretation was performed by HS, WT, MF, and SM. The manuscript was written by SM and edited by HS, WT, MF, and AS. All authors contributed to the article and approved the submitted version.

## Funding

This work was supported by funding from Bowel Research UK and Cancer Research UK (C16420/A18066); Rosetrees Trust (M492; M492-F1).

## Conflict of Interest

The authors declare that the research was conducted in the absence of any commercial or financial relationships that could be construed as a potential conflict of interest.

## Publisher’s Note

All claims expressed in this article are solely those of the authors and do not necessarily represent those of their affiliated organizations, or those of the publisher, the editors and the reviewers. Any product that may be evaluated in this article, or claim that may be made by its manufacturer, is not guaranteed or endorsed by the publisher.

## Supplementary Material

The Supplementary Material for this article can be found online at: https://www.frontiersin.org/articles/10.3389/fonc.2022.880552/full#supplementary-material

Supplementary Figure 1 FDA-Compound screen on RKO Ctrl wtAPC and APC mutant cells**(A)** Schematic showing the screen layout. The compound library was aliquoted over 14 x 96 well plates. On day 1 cells RKO Control*1 and APCmut*2 cells were plated, followed by drug treatment on day 2 and day 4. Then on day 6 cell viability was measured using CTG. **(B)** The Z score values were plotted for each compound in the screen for both cells lines (RKO Control*1 and APCmut*2 cells) to illustrate the spread of data. Data represnts results are from one screen replicate. RKO Control*1 are shown in red and APCmut*2 cells are shown in blue.

Supplementary Figure 2 Statin treatment decreases cytoplasmic β-catenin in APC-mutated cells**(A)** RKO Control*1, Control*2, APCmut*1 and APCmut*2 cells were treated with either vehicle (0.01% DMSO) or 4 μM lovastatin for 72 hours before fixing. Cells were then incubated with anti-β-catenin antibody and visualised via confocal microscopy. Total number of cells with cytoplasmic-localization of β-catenin were quantified and expressed as a % over total number of DAPI-stained cells. **(B)** RKO Control*1 and APCmut*1 cells were treated with either vehicle (0.01% DMSO), 4 μM lovastatin or 1 μM EHT1864, alone or in combination for 72 hours before fixing. Cells were then incubated with anti-β-catenin antibody and visualised via confocal microscopy. Total number of cells with cytoplasmic-localization of β-catenin were quantified and expressed as a % over total number of DAPI-stained cells. **p≤0.005. **(C)** Quantification of replicates of western blots (representative shown in **Figure 6B**) detecting levels of unphosphorylated β-catenin, normalised to β-actin. *p≤0.005; ***p≤0.0005.
