# Supplementary figures and images for: Statin Treatment as a Targeted Therapy for APC-Mutated Colorectal Cancer
Source: Front Oncol. 2022 May 30;12:880552. doi: 10.3389/fonc.2022.880552 (PMC9197185; doi:10.3389/fonc.2022.880552)

# SUPPLEMENTARY FIGURE 1

A

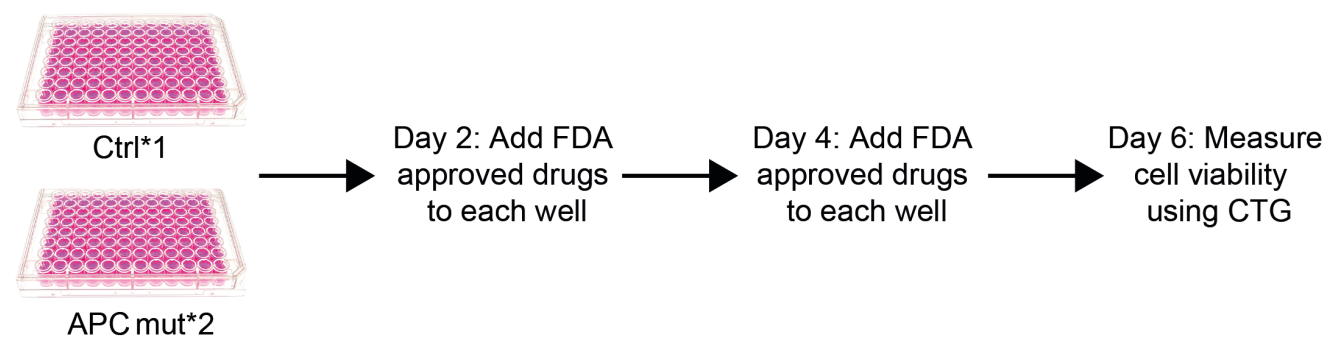

B

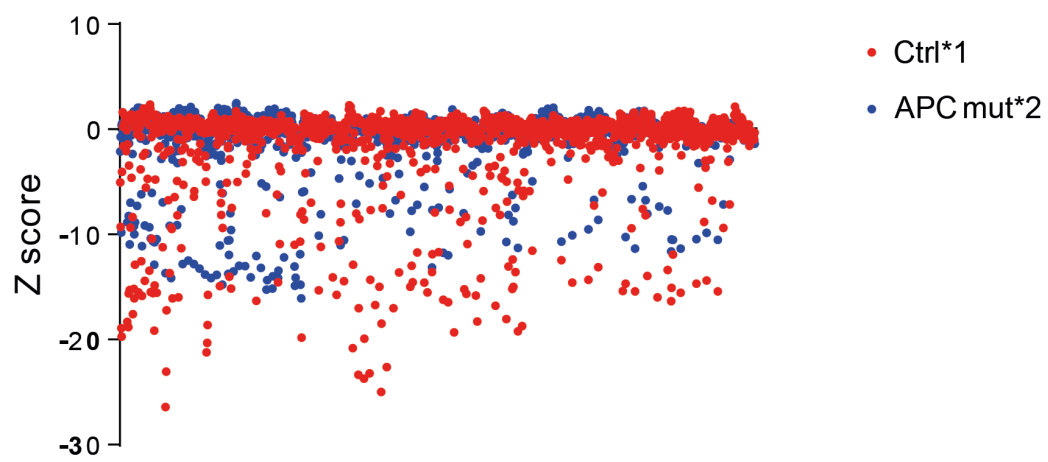

Supplement: Supplementary Figure 1 — FDA-Compound screen on RKO Ctrl wtAPC and APC mutant cells(A) Schematic showing the screen layout. The compound library was aliquoted over 14 x 96 well plates. On day 1 cells RKO Control*1 and APCmut*2 cells were plated, followed by drug treatment on day 2 and day 4. Then on day 6 cell viability was measured using CTG. (B) The Z score values were plotted for each compound in the screen for both cells lines (RKO Control*1 and APCmut*2 cells) to illustrate the spread of data. Data represnts results are from one screen replicate. RKO Control*1 are shown in red and APCmut*2 cells are shown in blue. [file DataSheet_1.pdf]

SUPPLEMENTARY FIGURE 2

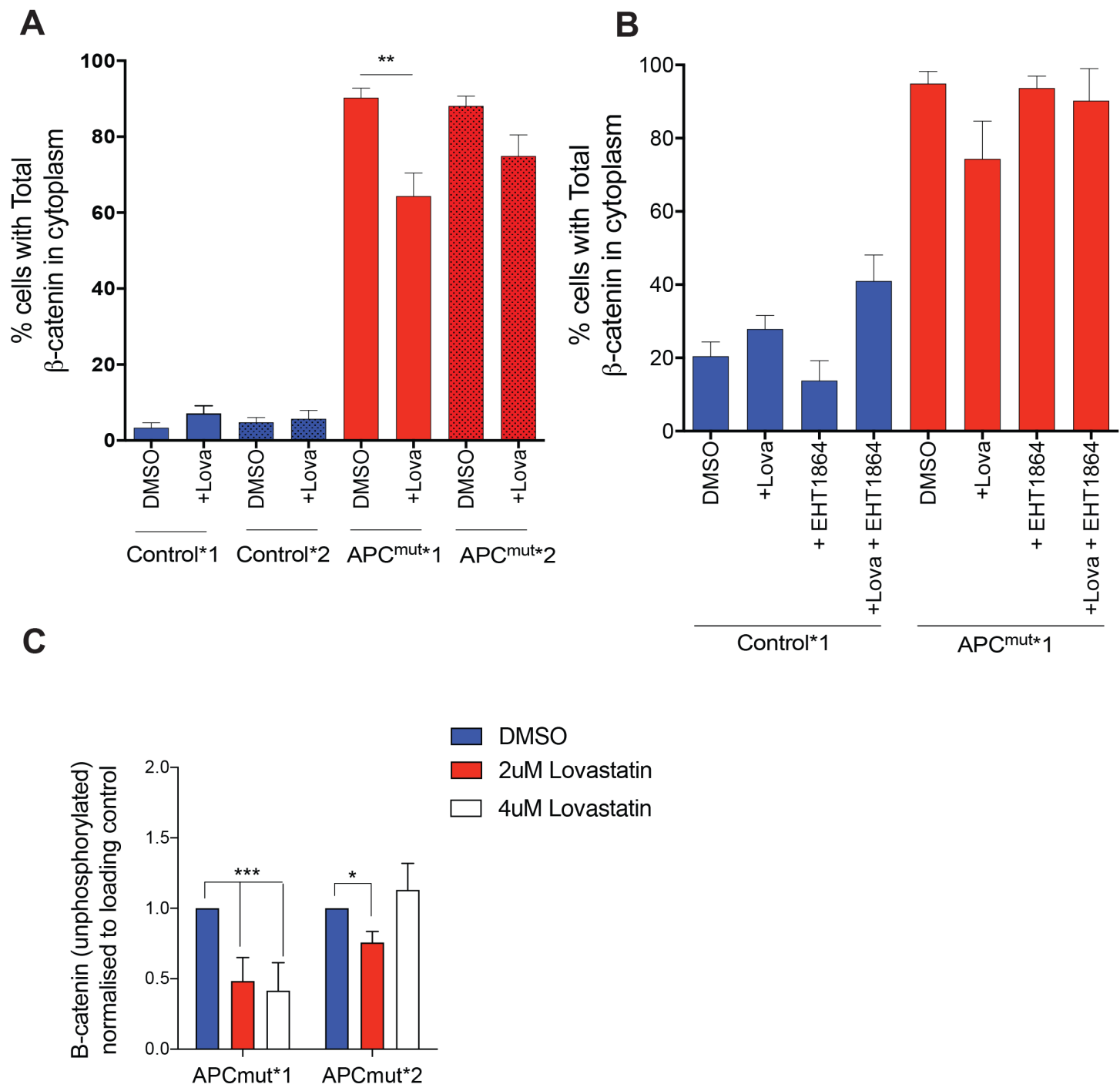

Supplement: Supplementary Figure 2 — Statin treatment decreases cytoplasmic β-catenin in APC-mutated cells(A) RKO Control*1, Control*2, APCmut*1 and APCmut*2 cells were treated with either vehicle (0.01% DMSO) or 4 μM lovastatin for 72 hours before fixing. Cells were then incubated with anti-β-catenin antibody and visualised via confocal microscopy. Total number of cells with cytoplasmic-localization of β-catenin were quantified and expressed as a % over total number of DAPI-stained cells. (B) RKO Control*1 and APCmut*1 cells were treated with either vehicle (0.01% DMSO), 4 μM lovastatin or 1 μM EHT1864, alone or in combination for 72 hours before fixing. Cells were then incubated with anti-β-catenin antibody and visualised via confocal microscopy. Total number of cells with cytoplasmic-localization of β-catenin were quantified and expressed as a % over total number of DAPI-stained cells. **p≤0.005. (C) Quantification of replicates of western blots (representative shown in Figure 6B) detecting levels of unphosphorylated β-catenin, normalised to β-actin. *p≤0.005; ***p≤0.0005. [file DataSheet_2.pdf]
